# Genetic Association between Different Metabolic Variants in *APOA5* and *PLIN1* in Type 2 Diabetes Mellitus among the Western Saudi Population: Case-Control Study

**DOI:** 10.3390/genes13071246

**Published:** 2022-07-14

**Authors:** Sherin Bakhashab, Tahani Alsulami, Amani M. T. Gusti, Steve Harakeh, Rajaa Al-Raddadi, Wissam A. Alwazani, Huda F. Alshaibi

**Affiliations:** 1 Department of Biochemistry, Faculty of Science, King Abdulaziz University, Jeddah 21589, Saudi Arabia; tahani14141993@gmail.com (T.A.); halshaibi@kau.edu.sa (H.F.A.); 2 Center of Excellence in Genomic Medicine Research, King Abdulaziz University, Jeddah 21589, Saudi Arabia; 3 Department of Medical Laboratory, Biochemistry, King Fahad Armed Forces Hospital, Jeddah 21159, Saudi Arabia; a_gusti2010@hotmail.com; 4 King Fahd Medical Research Center, King Abdulaziz University, Jeddah 21589, Saudi Arabia; sharakeh@gmail.com; 5 Yousef Abdul Lateef Jameel Chair of Prophetic Medicine Application, Faculty of Medicine, King Abdulaziz University, Jeddah 21589, Saudi Arabia; 6 Department of Community Medicine, Faculty of Medicine, King Abdulaziz University, Jeddah 21589, Saudi Arabia; saudiresearcher@yahoo.com; 7 Department of Biological Sciences, Faculty of Science, University of Jordan, Amman 11953, Jordan; wissamalwazani@gmail.com

**Keywords:** type 2 diabetes mellitus, single nucleotide polymorphism, apolipoprotein A5, perilipin 1

## Abstract

Type 2 diabetes mellitus (T2DM) is a multifactorial disease with a high global incidence. Hypertriglyceridemia is a major risk factor for both cardiovascular disease and T2DM. In this study, we determined the allele and genotype frequencies of apolipoprotein A5 (*APOA5*) single nucleotide polymorphism (SNP) rs662799 and perilipin 1 (*PLIN1*) SNPs rs894160, rs6496589, and rs1052700 and evaluated their association with T2DM risk in western Saudis. Only rs6496589 was found to be significantly associated with T2DM risk. We determined the risk allele for each SNP based on relative risk, and found that the G allele of rs662799, T allele of rs894160, G allele of r6496589, and T allele of rs1052700 correlated with T2DM risk. The effect of each SNP on T2DM risk and five of its clinical phenotypes was explored using multiple logistic regression. We found significant correlations between the C/G and G/G genotypes of rs6496589 and T2DM risk in the unadjusted model, whereas G/G was the only genotype that correlated with the risk of T2DM in the adjusted model. There was no significant correlation between rs662799, rs894160, and rs1052700 genotypes and T2DM risk. In conclusion, we have identified novel risk alleles and genotypes that contribute to genetic risk for T2DM in the western Saudi population.

## 1. Introduction

Type 2 diabetes mellitus (T2DM) is a major public health concern with a high incidence globally. Approximately 463 million adults have diabetes, according to the latest data published by the International Diabetes Federation [1]. This number is projected to increase to 700 million by 2045 if the comorbidity of diabetes is not well controlled [1]. A recent systematic analysis of 21 studies found that 32.8% of Saudis have T2DM, and its prevalence is predicted to increase to 40.37% by 2025 and 45.36% by 2030 [2].

Various studies have established that T2DM is a complex polygenic disorder determined by genetic, environmental, and behavioral factors [3]. Genetic variations play an important role in the development of T2DM, and over the past decade, genome-wide association studies (GWAS) have identified 143 single nucleotide polymorphisms (SNPs) associated with T2DM [4].

Hypertriglyceridemia is a key indicator of risk for cardiovascular disease and T2DM [5]. The apolipoprotein (*APO*) family is a cluster of genes consisting of four isoforms (APOA1/A4/C3/A5) located on chromosome 11q23 [6]. *APOA5* is primarily expressed in hepatocytes and secreted into the plasma, where it modulates triglyceride (TG) metabolism by binding to lipoprotein lipase to enhance TG catabolism and reduce very low-density lipoprotein production [7]. Insulin has been shown to inhibit *APOA5* promoter activity, potentially explaining the association between hypertriglyceridemia and hyperinsulinemia [8]. Therefore, variations in *APOA5* may contribute to the development and severity of T2DM. A meta-analysis of 19 studies found that SNP rs662799 upstream of *APOA5* is significantly associated with the incidence of diabetes among Asians. Individuals homozygous for the G allele had a 1.57-fold higher risk of developing T2DM than those homozygous for the A allele [9]. However, no significant association was detected between rs662799 and T2DM in Europeans [9].

Another important metabolic gene is perilipin 1 (*PLIN1*), a well-known member of the *PLIN* family located on chromosome 15q26 [10,11]. It has an essential role in lipid homeostasis, potentially blocking lipase-sensitive hormones, which have an important role in lipid breakdown in the basal state, leading to increased triglyceride storage through reduced triglyceride hydrolysis [12]. Thus, any defect in this mechanism can lead to obesity and complications such as T2DM [13]. A preliminary study in the Spanish population found that the intronic SNP rs894160 in *PLIN1* is a key factor in weight loss resistance [14]. Moreover, it has been found to be linked, either independently or synergistically, with other genes contributing to T2DM risk in Taiwanese individuals [15]. However, there was no direct association between *PLIN1* SNPs rs894160 and rs1052700 and diabetic risk in the Chinese, despite significant associations with central obesity, which can lead to T2DM [16]. Moreover, while Chinese individuals homozygous for the G allele at the *PLIN1* missense SNP rs6496589 had higher rates of lipolysis than those homozygous for the C allele, the G/G genotype was associated with lower waist circumference [17].

In this population study, we sought to determine the effect of SNPs in *APOA5* (rs662799) and *PLIN1* (rs894160, rs6496589, and rs1052700) on the risk for T2DM in the western Saudi population. Our findings fill a crucial knowledge gap on the contribution of these variants to genetic risk for T2DM in Saudis in the western region of Saudi Arabia.

## 2. Materials and Methods

### 2.1. Study Design

This case-control study was conducted between 2016 and 2018 in the Jeddah Care Center for Diabetes and Hypertension and the Endocrinology and Diabetes Center outpatient clinic at King Fahad Armed Forces Hospital in Jeddah, Saudi Arabia. This study was approved by the Ministry of Health (approval number A00363) and the institutional review board at the King Fahd Armed Forces Hospital (approval number REC 201) and conducted according to the Declaration of Helsinki. Written informed consent was obtained from all participants prior to sample collection.

The total sample size was 406 participants following the exclusion of patients with other chronic diseases, such as cardiovascular disease and metabolic syndrome. We estimated the power of our study to be 95% based on a sample size of 406 subjects and an odds ratio (OR) of 1.5 for the studied SNPs using the G*Power software 3.1 [18,19]. We successfully recruited 223 T2DM patients and 183 controls. All participants in the current study were identified as Saudi based on self-reported ethnicity, which should present itself as a homogenous sample set genetically. This homogeneous ancestry will decrease the type 2 error rate [20]. The following clinical parameters were recorded for all participants: age, sex, body weight, height, and body mass index (BMI). The following biochemical parameters were measured: hemoglobin A1c (HbA1c), fasting blood glucose (FBS), TG, cholesterol, and low-density lipoprotein (LDL).

### 2.2. Genotyping 

Genomic DNA was isolated from whole blood using the QIAamp Blood Maxi Kit (Qiagen, Hilden, Germany) according to the manufacturer’s recommended protocol. All samples were genotyped for *APOA5* SNP rs662799 (assay ID: C___2310403_10), and *PLIN1* SNPs rs6496589 (C__30373512_10), rs894160 (C___8722593_10), and rs1052700 (C___8722587_10), using TaqMan SNP Genotyping Assays (Thermo Fisher Scientific, Waltham, MA, USA). Allelic PCR products were analyzed using QuantStudio 12K Flex Real-Time PCR System (Thermo Fisher Scientific). The reaction mixture was prepared using TaqMan^®^ GTXpress^TM^ Master Mix (Thermo Fisher Scientific) according to the manufacturer’s recommendations. The real-time PCR was run under the following conditions: polymerase activation at 95 °C for 10 min, and 40 cycles of 15 s at 95 °C and 1 min at 60 °C in MicroAmp Optical 96-well reaction plates (Thermo Fisher Scientific).

### 2.3. Statistical Analysis 

Quantitative data are presented as mean ± standard deviation (SD), while qualitative data are presented as frequencies and percentage. For normally distributed data, a Student’s *t*-test was used to compare two unmatched groups, and a *p*-value < 0.05 was considered statistically significant. Genotypes and allelic frequencies were calculated within the cohort population to determine the significant differences between the T2DM patients and control group. The association between the studied SNPs and T2DM was determined based on Pearson’s chi-square test. Furthermore, the relative risk for alleles was calculated to determine the risk allele in each SNP. Allele frequencies in patients and controls were compared using the Chi-square test. A binary logistic regression was performed to estimate the association between T2DM and the studied SNPs genotypes. To adjust for other variables, multiple logistic regression was used. The adjusted and unadjusted odds ratio (OR) were presented together with a 95% confidence interval (95% CI) Data analyses were performed using IBM SPSS software version 25 (SPSS^TM^ Inc., Armonk, NY, USA).

## 3. Results

### 3.1. The Association of APOA5 and PLIN1 Variants with T2DM Risk

The clinical parameters recorded for all participants are summarized in Table 1. The genotype frequencies of *APOA5* SNP rs662799 and *PLIN1* SNPs rs894160, rs6496589, and rs1052700 are provided in Table 2. We assessed the association of each SNP with T2DM using Pearson’s chi-squared test. We found a significant association between *PLIN1* SNP rs496589 and susceptibility to T2DM (*p* < 0.001). However, no significant associations were observed between T2DM risk and *APOA5* SNP rs662799 and *PLIN1* SNPs rs894160 and rs1052700.

### 3.2. Relative Risk of T2DM for Alleles in APOA5 and PLIN1 SNPs

The allele frequencies of *APOA5* SNP rs662799 and *PLIN1* SNPs rs894160, rs6496589, and rs1052700 are provided in Table 3. To determine the risk allele for each SNP, we calculated their relative risk (RR), and found that G was the risk allele for *APOA5* SNP rs662799. This allele was associated with a RR for T2DM of 1.02 (Table 3). For *PLIN1* SNP rs894160, T was the risk allele with a RR for T2DM of 1.13, while G was the risk allele for *PLIN1* SNP rs6496589 with a RR for T2DM of 1.72, and T was the risk allele for *PLIN1* SNP rs1052700 with a RR for T2DM of 1.06.

### 3.3. The Association between T2DM, APOA5 and PLIN1 SNPs and Clinical Parameters

To explore genetic risk for T2DM and its clinical parameters, we assessed the influence of each genotype for *APOA5* SNP rs662799 and *PLIN1* SNPs rs894160, rs6496589, and rs1052700 using binary logistic regression. No significant association between *APOA5* SNP rs662799, *PLIN1* SNP rs894160 and PLIN SNP rs1052700 genotypes and T2DM risk were observed (Table 4). However, a significant association was observed between *PLIN1* SNP rs6496589 and T2DM. Individuals carrying the C/G or G/G genotype were significantly more likely to have T2DM (OR = 3.0 with *p* = 0.009 and OR = 24.0 with *p* = 0.002, respectively) based on an unadjusted binary logistic regression model (Table 4). Hence, the G allele of SNPs rs6496589 is a potential risk allele for T2DM, and carrying one G allele is sufficient to increase the risk for T2DM. In the multiple logistic regression model, G/G genotype showed the highest association with the risk of T2DM (OR = 15.2, *p* = 0.02, Table 5). Subsequently, LDL (OR = 2.7, *p* = 0.043), and age (OR = 1.1, *p* < 0.001) were significantly associated with the risk of T2DM (Table 5). Therefore, in the current logistic regression model, the association of G/G genotype for *PLIN1* SNP rs6496589 and other factors including age and LDL increase the risk of T2DM independently.

## 4. Discussion

T2DM is considered one of the most common chronic multifactorial diseases worldwide. The situation is more complex in Saudi Arabia, where one in five people are diagnosed with chronic, progressive T2DM. This is double the global rate for T2DM, and it has serious economic and financial implications in Saudi Arabia [2]. Several clinical and epidemiological studies have casually linked lipid metabolism dysfunction with risk for T2DM [21,22]. However, the findings of these studies have proven controversial [23].

In the current study, we assessed the impact of SNPs in the *PLINI* and *APOA5* genes on susceptibility for T2DM, selecting each based on the inhibitory effect of insulin in TG catabolism [9,24,25,26,27]. To the best of our knowledge, this is the first study to report an association between these SNPs and dysglycemia disorders in Saudis. It is well known that *APOA5* SNP rs662799 is strongly associated with metabolic syndromes and elevated plasma TG [23]. Moreover, several studies in different ethnic groups have reported a strong association between *APOA5* -1131T/C polymorphism and the development of metabolic syndrome [24,25]. We thus investigated the relationship between *APOA5* SNP rs662799 and T2DM in Saudis.

In our cohort, the frequency of major allele A for *APOA5* SNP rs662799 was 0.86 in the T2DM group and 0.87 in the control group, which is highly similar to its frequency (0.84) in the 1000 Genomes Project data global population (https://www.ncbi.nlm.nih.gov/snp/rs662799, accessed on 8 February 2022). No significant associations were observed between SNPs in *APOA5* and RR for T2DM. This finding accords with a meta-analysis of Europeans that found the *APOA5* -1131T/C polymorphism was not associated with T2DM risk [9].

The second gene we studied was *PLIN1* to investigate the associations between *PILN* polymorphism and glucose metabolism. It is well known that *PLIN1* is a key regulatory protein for adipose tissue metabolism and that variation in this gene is associated with diabetic risk [28]. The function of perilipins is to prevent lipolysis in basal conditions, which promotes fat deposition [28,29]. We made a number of significant observations when we genotyped the three *PLIN1* SNPs in our participants. While SNPs rs894160 and rs1052700 were not significantly associated with T2DM, SNP rs6496589 was and can be explained by the relationship between central obesity and the rate of lipolysis [17]. In line with our results, rs894160 polymorphisms of *PLIN1* was not a major genetic determinant of T2DM in a random sample of a French cohort [30]. In addition, no significant association between rs894160, rs1052700 SNPs and the risk of T2DM was detected among Chinese adults [16].

For the *PLIN1* gene, we explored the major allele frequencies for all three SNPs considered: rs894160, rs6496589, and rs1052700. Their frequencies were 0.63, 0.84, and 0.59 in the T2DM group, respectively, and 0.70, 0.97, and 0.62 in the control group. Conversely, the minor allele frequencies were 0.36, 0.16, and 0.41 in the T2DM group, respectively, and 0.30, 0.03, and 0.38 in the control group. The observed frequencies are more similar to the US population than the Spanish population due to ethnic variation [13,28].

Our results highlight a remarkable association between *PLIN1* SNP rs6496589 and T2DM. Patients carrying a C/G or G/G genotype were 3.0 and 24.0 times more susceptible to T2DM than the control group, respectively, in the unadjusted model. Therefore, we considered the G allele as a potential risk allele for T2DM. These observations can be explained by the association between *PLIN1* polymorphism and increased lipolysis leading to the accumulation of fatty acids, which adversely affects insulin sensitivity [13,17,31]. Data from different studies indicate that the genetic effect of *PLIN1* depends on obesity and adipokine secretion, which can negatively impact adipose tissue endocrine function and lead to diabetes [13,31,32,33].

There is only limited data on the relationship between *PLIN1*, adipose tissue endocrine function, and metabolic disorders. Additional studies are required to elucidate the underlying mechanism, accounting for gender, obesity status, ethnic origin, and population size, to eliminate potential biases that may confound results and hinder the identification of causal genetic variants.

In summary, *PLIN1* gene SNP rs6496589 has a substantial role in metabolic disorders, insulin resistance, and the development of T2DM.

## Figures and Tables

**Table 1:** Clinical characteristics of T2DM and control subjects.

Variable	Control Sample (*n* = 183)	Diabetic Sample (*n* = 223)	*p*-Value
Gender (M/F)	150/33	95/118	<0.001 ***
Age (years)	38.8 ± 13.3	52.6 ± 16.9	<0.001 ***
BMI (kg/m^2^)	27.8 ± 5.3	29.7 ± 6	0.001 **
Obesity (yes/no)	125/58	170/53	0.075
HbA1c (%)	5.5 ± 0.5	7.2 ± 1.9	<0.001 ***
HbA1c >6 (yes/no)	16/167	150/71	<0.001 ***
FBS (mmol/L)	5.6 ± 1	8.5 ± 3.7	<0.001 ***
Triglycerides (mmol/L)	1.3 ± 1	1.5 ± 2.3	0.193
Cholesterol (mmol/L)	4.7 ± 1	4.8 ± 1.1	0.724
LDL (mmol/L)	2.8 ± 0.9	3.1 ± 1.5	0.040 *

Quantitative data are presented as mean ± SD, and qualitative data are presented as frequencies and percentage. All *p*-values were calculated using Student’s unpaired *t*-test for continuous variables and a chi-squared test for categorical variables. Obese individuals were defined as those with a BMI ≥ 25. BMI: Body mass index, FBS: Fasting blood glucose, HbA1c: Glycated hemoglobin, LDL: Low-density lipoprotein. * *p* < 0.05, ** *p* < 0.01, *** *p* < 0.001.

**Table 2:** Associations identified between *APOA5* and *PLIN1* SNPs and T2DM risk.

SNP	Genotype	Diabetic Sample	Control Sample	*p*-Value
*APOA5* rs662799	A/A A/G G/G	164 (74.9%) 49 (22.4%) 6 (2.7%)	137 (75.7%) 39 (21.5) 5 (2.8%)	0.980
*PLIN1* rs894160	C/C C/T T/T	90 (40.7%) 101(45.7) 30 (13.6)	88 (48.1) 79 (43.2%) 16 (8.7%)	0.180
*PLIN1* rs6496589	C/C C/G G/G	174 (78.7%) 23 (10.4%) 24(10.9%)	174 (95.1%) 8 (4.4%) 1 (0.5%)	<0.001 ***
*PLIN1* rs1052700	A/A A/T T/T	85 (38.6%) 89 (40.5%) 46 (20.9%)	73 (39.9%) 81 (44.3%) 29 (15.8%)	0.415

Data are presented as frequencies, and p-values were calculated using Pearson’s chi-squared test. All *p*-values < 0.05 were considered statistically significant. *** *p* < 0.001.

**Table 3:** Relative risk for T2DM from alleles in *APOA5* and *PLIN1 SNPs*.

SNP	Allele	Allele Frequency (T2DM)	Allele Frequency (Control)	RR	OR	*p*-Value
*APOA5* rs662799	G A	61 (13.9%) 377 (86.1%)	49 (13.50%) 313 (86.5%)	1.02	1.03	0.873
*PLIN1* rs894160	T C	161 (36.4%) 281(63.6%)	111 (30.3%) 255 (69.7%)	1.13	1.32	0.068
*PLIN1* rs6496589	G C	71 (16.1%) 371 (83.9%)	10 (2.7%) 356 (97.3%)	1.72	6.81	<0.001 ***
*PLIN1* rs1052700	T A	181 (41.1%) 259 (58.9%)	139 (38.0%) 227 (62.0%)	1.06	1.14	0.362

Data are presented as frequencies, and *p*-values calculated using a 2 × 2 contingency chi-square test with Yate’s continuity correction. All *p*-values < 0.05 were considered statistically significant. OR: Odds ratio, RR: Relative risk. *** *p* < 0.001.

**Table 4:** Association between the risk of T2DM and each SNP genotype.

SNP	Genotype	B	*p*-Value	OR	95% C.I. for OR	
					Lower	Upper
*APOA5* rs662799	A/G	−0.046	0.943	0.955	0.271	3.364
	G/G	−0.048	0.843	0.953	0.591	1.536
*PLIN1* rs894160	C/T	0.199	0.348	1.220	0.805	1.850
	T/T	0.595	0.084	1.812	0.923	3.558
*PLIN1* rs6496589	C/G	1.099	0.009 **	3.000	1.312	6.861
	G/G	3.178	0.002 **	24.000	3.211	179.368
*PLIN1* rs1052700	A/T	−0.309	0.279	0.734	0.419	1.285
	T/T	−0.367	0.194	0.693	0.398	1.205

*p*-values were calculated using binary logistic regression. A/A, C/C, C/C and A/A are the reference genotypes for *APOA5* SNP rs662799 and *PLIN1* SNPs rs894160, rs6496589, and rs1052700, respectively, and T2DM was the dependent variable. B: β coefficient; OR, odds ratio; CI, confidence interval. ** *p* < 0.01.

**Table 5:** Adjusted odd ratio for the association between T2DM, *PLIN1* SNP rs6496589 and clinical variables.

Variables	B	*p*-Value	OR (95% C.I.)
rs6496589 (C/G)	0.5	0.72	1.6 (0.12–23.1)
rs6496589 (G/G)	2.7	0.02 *	15.2 (1.5–154.5)
Age (year)	0.1	<0.001 ***	1.1 (1.08–1.2)
BMI (kg/m^2^)	0.05	0.20	1.05 (0.9–1.1)
Triglycerides (mmol/L)	0.09	0.7	1.09 (0.7–1.7)
Cholesterol (mmol/L)	−1.0	0.051	0.4 (0.2–1.0)
LDL (mmol/L)	1.0	0.043 *	2.7 (1.03–6.9)

All *p*-values were calculated using multiple logistic regression. C/C is the reference genotype and T2DM was the dependent variable. B: β coefficient; BMI, body mass index; OR, odds ratio; CI, confidence interval. * *p* < 0.05, *** *p* < 0.001.

## Data Availability

Not applicable.
